# Derivatives of 2-aminobenzimidazole potentiate ASIC open state with slow kinetics of activation and desensitization

**DOI:** 10.3389/fphys.2023.1018551

**Published:** 2023-01-12

**Authors:** Konstantin K Evlanenkov, Margarita S Komarova, Mikhail Y Dron, Maxim V Nikolaev, Olga N Zhukovskaya, Nataliya A Gurova, Denis B Tikhonov

**Affiliations:** ^1^ I.M. Sechenov Institute of Evolutionary Physiology and Biochemistry, St. Petersburg, Russia; ^2^ Research Institute of Physical and Organic Chemistry, Southern Federal University, Rostov-on-Don, Russia; ^3^ Department of Pharmacology and Bioinformatics, Volgograd State Medical University, Volgograd, Russia

**Keywords:** ion channels, modulation, kinetics of action, structure-function relationships, pharmacology

## Abstract

The pharmacology of acid-sensitive ion channels (ASICs) is diverse, but potent and selective modulators, for instance for ASIC2a, are still lacking. In the present work we studied the effect of five 2-aminobenzimidazole derivatives on native ASICs in rat brain neurons and recombinant receptors expressed in CHO cells using the whole-cell patch clamp method. 2-aminobenzimidazole selectively potentiated ASIC3. Compound Ru-1355 strongly enhanced responses of ASIC2a and caused moderate potentiation of native ASICs and heteromeric ASIC1a/ASIC2a. The most active compound, Ru-1199, caused the strongest potentiation of ASIC2a, but also potentiated native ASICs, ASIC1a and ASIC3. The potentiating effects depended on the pH and was most pronounced with intermediate acidifications. In the presence of high concentrations of Ru-1355 and Ru-1199, the ASIC2a responses were biphasic, the initial transient currents were followed by slow component. These slow additional currents were weakly sensitive to the acid-sensitive ion channels pore blocker diminazene. We also found that sustained currents mediated by ASIC2a and ASIC3 are less sensitive to diminazene than the peak currents. Different sensitivities of peak and sustained components to the pore-blocking drug suggest that they are mediated by different open states. We propose that the main mechanism of action of 2-aminobenzimidazole derivatives is potentiation of the open state with slow kinetics of activation and desensitization.

## 1 Introduction

Acid-sensitive ion channels (ASICs), which are activated by extracellular protons, are widely expressed in the central and peripheral nervous system. They are involved in a large number of normal physiological processes and in various pathologies (Mango and Nistico, 2021; Storozhuk et al., 2021). ASIC3 and ASIC1b subunits are found mainly on the periphery, whereas ASIC1a and ASIC2 subunits have been detected in both the central and peripheral nervous system (Kellenberger and Schild, 2015). ASIC2b cannot form a functional channel on its own. Homotrimer ASIC2a is less sensitive to pH than ASIC1a, ASIC1b, and ASIC3 (Hesselager et al., 2004). Numerous studies have been devoted to the structure, pharmacology, and function of ASIC1a, whereas ASIC2a is less well studied despite the unique roles they play (Sivils et al., 2022). Many atomic-scale structures in different functional states and in complex with drugs are available for ASIC1a (Jasti et al., 2007; Dawson et al., 2012; Baconguis et al., 2014; Liu et al., 2021) but not for ASIC2a.

The pharmacology of ASICs is quite diverse (Osmakov et al., 2014; Baron and Lingueglia, 2015; Cristofori-Armstrong and Rash, 2017; Rash, 2017; Vullo and Kellenberger, 2020). ASIC-targeting agents include inorganic ions, natural and synthetic small molecules, and peptide toxins from various poisons. They target the proton recognition site(s), the ion channel pore, and several modulating sites. Many powerful and selective modulators have been identified for ASIC1a and ASIC3, but selective ASIC2a-targeting agents are rare. Extracellular Zn^2+^ has a potentiating effect at micromolar concentrations on ASIC2a-containing channels (Baron et al., 2001) and an inhibitory effect at nanomolar concentrations on both homomeric ASIC1a and heteromeric ASIC1a and ASIC2a channels (Chu et al., 2004). The potentiating effect of Zn^2+^ is due to a shift in the pH dependence of the ASIC1a+2a activation from pH_50_ 5.5 to 6.0. Systematic mutagenesis of 10 extracellular histidines in ASIC2a led to the identification of two residues (His-162 and His-339) that are necessary for the potentiating effect of Zn^2+^(Baron et al., 2001).

In our previous work, we found selective potentiation of ASIC2a homomers by memantine, IEM-1921, and IEM-2115 (Tikhonova et al., 2015; Nagaeva et al., 2016a). The clinically used antidepressants desipramine, amitriptyline, fluoxetine, and atomoxetine, and the neuroleptic chlorpromazine also potentiate both ASIC1a and ASIC2a, whereas tianeptine has been described as a selective inhibitor of ASIC2a (Nikolaev et al., 2019). However, the activity of these compounds is rather weak. Thus, active and selective modulators of ASIC2a homomers and ASIC2a-containing heteromers are lacking, which complicates physiological studies of their specific functions.

**FIGURE 1:**
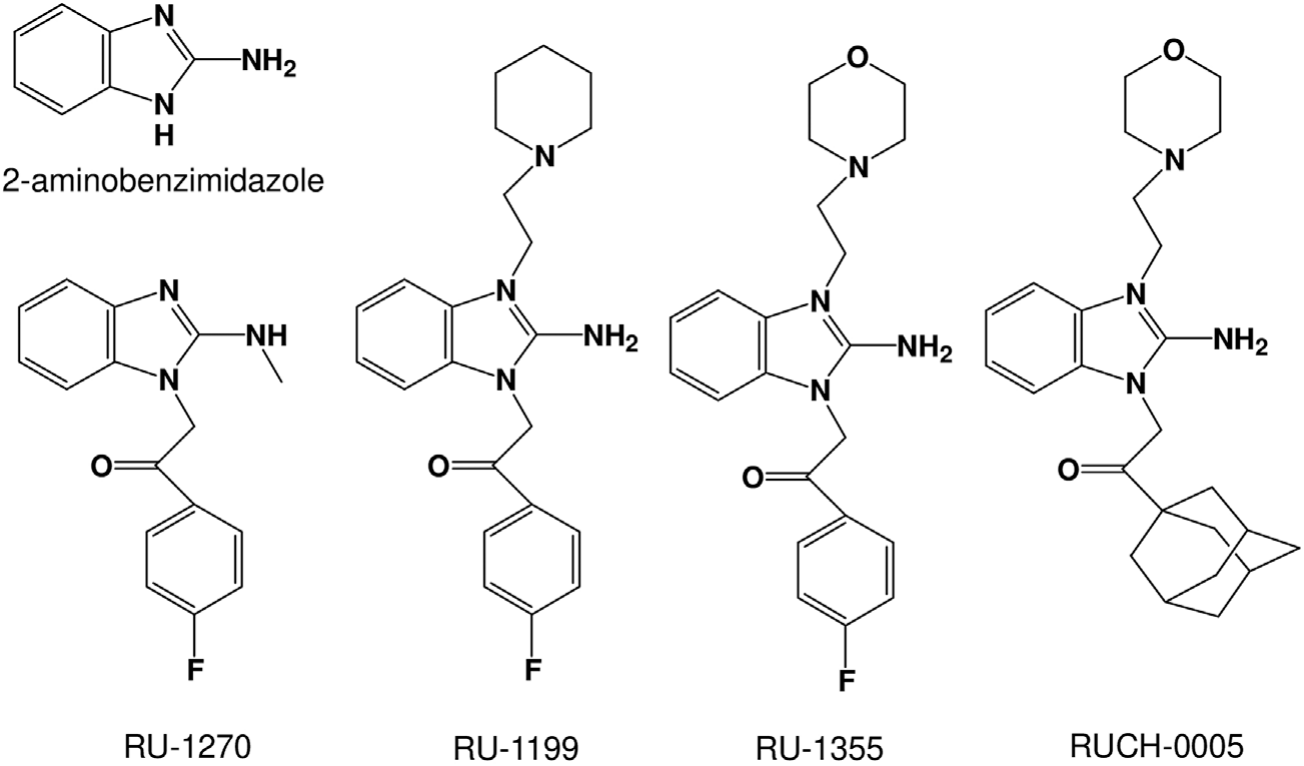
Chemical structures of the 2-aminobenzimidazole derivatives studied.

In the present study, we focused on finding new ASIC modulators, bearing in mind that they can be selective ASIC2a modulators. We selected several derivatives of 2-aminobenzimidazole (Figure 1), as the structure of 2-aminobenzimidazole resembles well-known ASIC modulators GMQ and amiloride. Previous study has shown that Ru-1355, a derivative of 2-aminobenzimidazole, exhibits inhibitory activity against the sodium-hydrogen exchanger NHE-1 (Spasov et al., 2016). Amiloride, the first drug described as an inhibitor of NHE-1 (Benos, 1982), is also known to block ASICs, suggesting that 2-aminobenzimidazole and its derivatives could be ASIC modulators.

We found that the 2-aminobenzimidazole selectively potentiates ASIC3. Compound Ru-1355 strongly enhanced responses of ASIC2a and caused moderate potentiation of native ASICs and heteromeric ASIC1a/ASIC2a. The most active compound, Ru-1199, caused the strongest potentiation of ASIC2a, but also potentiated native ASICs, ASIC1a and ASIC3. Analysis of the mechanisms of action suggests that the compounds potentiate a specific open state with slow kinetics, which is weakly sensitive to ASIC pore blocker diminazene.

## 2 Materials and methods

### 2.1 Native receptors

The experiments were conducted in accordance with the Rules of the Committee for the Care and Use of Animals of the I.M. Sechenov Institute of Evolutionary Physiology and Biochemistry of the Russian Academy of Sciences, which is fully compatible with the directives of the Council of the European Community 86/609/EEC. Outbred male Wistar rats, aged 12–17 days and weighing 25–35 g, were obtained from a local animal facility. The rats were anesthetized with urethane and then decapitated. The brain was quickly extracted and cooled to 2–4°C in an ice bath. Transverse prefrontal cortex slices (250 µm thick) were prepared using a vibratome (Campden Instruments Ltd. Loughborough, UK) and stored in a solution containing (in mM): NaCl 124, KCl 5, CaCl_2_ 1.3, MgCl_2_ 2.0, NaHCO_3_ 26, NaH_2_PO_4_ 1.24, and d-glucose 10, aerated with carbogen (95% O_2_, 5% CO_2_). Neurons were isolated from brain slices using vibrodissociation method (Vorobjev, 1991). We used pyramidal neurons from the second and third layers of the medial prefrontal cortex, striatal interneurons and hippocampal CA1 pyramidal neurons. ASICs in these neurons were characterized previously (Sherwood et al., 2012; Kellenberger and Schild, 2015). The neurons were identified by their morphological characteristics.

### 2.2 Recombinant receptors

CHO cells were cultured at 37°C in a humidified atmosphere of 5% CO_2_. Cells were maintained with standard culture conditions (Dulbecco’s modified Eagle’s medium [DMEM] + 12% fetal bovine serum + gentamicin (50 μg/ml). Plasmids encoding ASIC subunits were transfected using Lipofectamine 2000 (Invitrogen, CA, United States) following the manufacturer’s transfection protocol. The cells were transfected with 0.5 μg rASIC1a, rASIC2a, or rASIC3 cDNA per 35 mm^2^ dish +0.5 μg green fluorescent protein (GFP). The experiments were carried out 48–72 h after transfection.

### 2.3 Electrophysiology

The whole-cell acid-evoked currents were recorded by a patch clamp technique at a holding potential of -80 mV using EPC-10 amplifier (HEKA Electronics, Lambrecht, Germany). The currents were filtered at 5 kHz and digitized at a sampling rate of 1 kHz using the PatchMaster software (HEKA Electronics, Lambrecht, Germany). All experiments were performed at room temperature (23–25°C). Standard chemicals were purchased from Sigma-Aldrich, Tocris Bioscience, and Chimmed. The 2-aminobenzimidazole derivatives studied here were synthesized by the Laboratory of Organic Synthesis, Research Institute of Physical and Organic Chemistry, Southern Federal University, Rostov-on-Don, Russian Federation. The initial solutions of the studied preparations were prepared using 100% DMSO (Sigma-Aldrich). The drug-containing solutions were made from the extracellular solution, and their pH values were adjusted (if needed) to the required values after addition of the drug. The final concentration of DMSO in working solutions was <1%. Patch pipettes (two to five MΩ) were made with a micropipette puller P-97 (Sutter Instruments, CA, United States). The extracellular solution contained (in mM): NaCl 143, KCl 5, MgCl_2_ 2, CaCl_2_ 2.5, d-glucose 18, HEPES 10, and MES 10 (pH was adjusted to 7.4 with NaOH). Acidic solutions were prepared from the extracellular solution by pH adjustment for the desired values with HCl. The pipette solution contained (in mM): CsF 100, CsCl 40, NaCl 5, CaCl_2_ 0.5, EGTA 5, and HEPES 10 (pH was adjusted to 7.2 with CsOH). For fast drug application, a micromanifold and the RSC-200 perfusion system (BioLogic Science Instruments, Claix, France) were used. In our setup, this system provides a solution exchange time of about 200 m (Nikolaev et al., 2019). To avoid the accumulation of drugs and pH changes in the experimental chamber, the solutions were removed using a suction system.

### 2.4 Data analysis and statistics

All values are presented as the mean ± standard deviation (SD) from at least five experiments (cells). The data on recombinant ASICs were collected from at least two (usually 3–4) transfections. The data on native ASICs were obtained from at least three (usually 4–5) animals. The significance of the effects was tested with a paired *t*-test (drug vs. control). For the data obtained from different cells, the unpaired test was used. For multiple data sets, ANOVA with Tukey’s post-test was used. The effects were considered significant at *p* < 0.05, based on at least 5 experiments. For statistical analysis and curve fitting Microcal Origin 9.0 was used. Concentration dependencies were approximated by Hill equation. The decay time constants (τ) were obtained by approximating the descending part of the responses using a single-exponential function.

## 3 Results

### 3.1 Control experiments

We estimated the effects of compounds shown in Figure 1 on native ASICs in rat pyramidal neurons from the second and third layers of the medial prefrontal cortex and on recombinant ASICs in the CHO expression system. In control experiments, acidification to pH 6.0 solutions did not produce detectable currents in non-transfected CHO cells or cells transfected by GFP only. In both cases pH 4.0 caused non-desensitizing inward currents of 29 ± 7 (n = 11) pA. These currents are negligible in comparison with the ASIC responses at this pH, which were above 1 nA. Application of the compounds at concentration of 100 μM in CHO cells without ASIC transfection did not evoke a response at pH 6.0 and did not modify responses to pH 4.0. Under neutral conditions (pH 7.4) the compounds did not evoke a response in neurons or in the CHO cells transfected with ASIC1a, ASIC2a, or ASIC3 plasmids. DMSO at the concentration used also did not evoke a response in any of the cell types.

### 3.2 Structure-activity relationships

The compounds studied are shown in Figure 1. They are derivatives of 2-aminobenzimidazole with various substitutions. The effects of potentiation and inhibition were assessed by activating the ASICs by acidification, which produced 30–50% of the maximum response (pH 6.5 for native ASICs and ASIC1a, pH 5.0 for ASIC2a and pH 6.85 for ASIC3). The duration of the application was 15 s for ASIC1a, ASIC3 and native ASICs, 80 s for ASIC2a, due to their different desensitization kinetics. The interval between applications was 30 s to ensure recovery after desensitization in all cases. The compounds were applied at a concentration of 100 μM simultaneously with activating acidifications.

**FIGURE 2:**
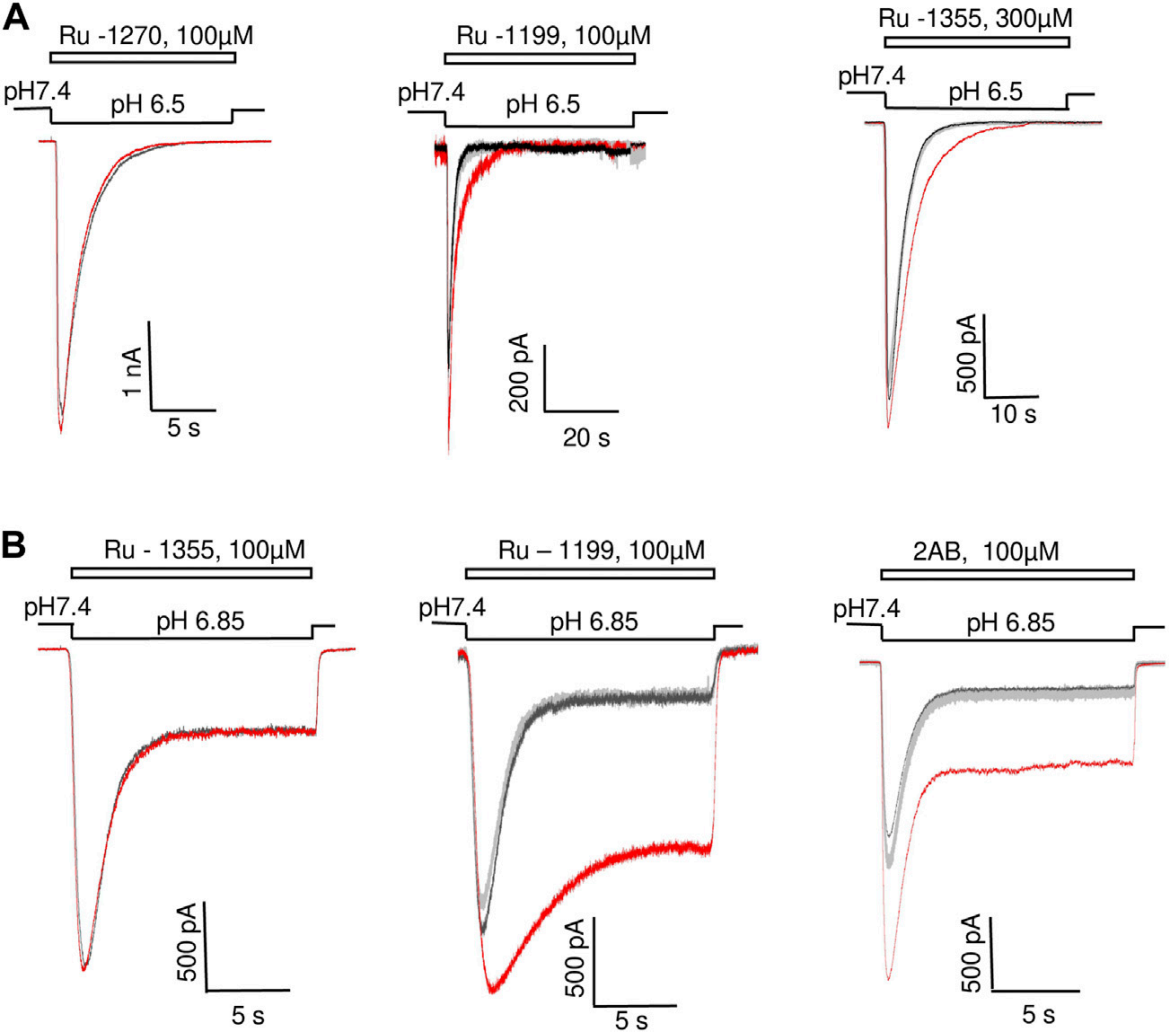
Action of 2-aminobenzimidazole derivatives on ASIC1a **(A)** and ASIC3 **(B)**. Responses in the control condition are shown in black; responses in the presence of the drug are shown in red; responses after drug washout are shown in gray. A, Ru-1270 is inactive. Ru-1199 potentiates the response amplitude and slows down the decay of the ASIC1a response. Ru-1355 (300 μM) does not significantly affect the response amplitude, but slows down the kinetics of desensitization. B, Ru-1355 is inactive, whereas Ru-1199 and 2-AB potentiate both the peak and steady-state components of the ASIC3 response.

The effects of drugs on peak currents are presented in Table 1. RUCH-0005 was virtually inactive. Ru-1270 demonstrated modest inhibition of native ASICs without significant effects on the recombinant receptors studied. 2-aminobenzimidazole selectively potentiated ASIC3. Ru-1355 had a potentiating effect on native ASICs and ASIC2a. The most active compound, Ru-1199, had a significant effect on ASIC1a and ASIC3. ASIC2a was also sensitive to 3 μM Ru-1355 and 1 μM Ru-1199 (see Table 1 and Figure 2). All effects were fully reversible.

**TABLE 1:** Activities of 2-aminobenzimidazole derivatives (drug vs. control).

Channel	RUCH-0005	Ru-1270	2-AB	Ru-1355	Ru-1199
ASIC3 (100 μM)	1.02 ± 0.04 n = 6	0.92 ± 0.10 n = 5	1.85 ± 0.33^a^ n = 7	1.00 ± 0.05 n = 5	1.36 ± 0.1^a^ n = 5
ASIC2a (3 μM)	0.99 ± 0.02 n = 6	1.01 ± 0.03 n = 8	1.01 ± 0.02 n = 5	1.25 ± 0.11^a^ n = 6	1.35 ± 0.12^a^ n = 5 (1 μM)
ASIC1a (100 μM)	0.95 ± 0.11 n = 5	1.01 ± 0.02 n = 5	0.96 ± 0.14 n = 5	1.01 ± 0.02 n = 5	1.31 ± 0.03^a^ n = 8
native ASIC (100 μM)	1.00 ± 0.18 n = 5	0.72 ± 0.11^a^ n = 5	1.00 ± 0.06 n = 5	1.75 ± 0.13^a^ n = 10	2.20 ± 0.33^a^ n = 5

^a^- the effect is statistically significant (paired *t*-test).

The compounds significantly affected the shape of responses. Potentiation of ASIC1a amplitude by Ru-1199 included slowing down of the response decay (*τ* = 2.1 ± 0.5, n = 7 in control and 3.6 ± 0.7, n = 5 in the presence of the drug, *p* < 0.05). As a result, during standard 15 s activation, the response did not desensitize completely. An additional experiment with prolonged (40s) activation demonstrated that desensitization was complete (Figure 2A, middle panel). Although the effect of Ru-1355 on the ASIC1a response amplitude was not pronounced, the compound caused a slight, but significant (decay time constant was increased by 1.28 ± 0.15, n = 5, *p* < 0.05), slowing of the desensitization. An additional test of Ru-1355 at a concentration of 300 μM revealed a stronger effect (1.73 ± 0.45, n = 5) on desensitization with a subtle effect on the amplitude (Figure 2A, right panel). The effects of 2-aminobenzimidazole and Ru-1199 on ASIC3 included potentiation of the peak and sustained components of the response (Figure 2B). The sustained current potentiation (Idrug/Icontr) was 4.7 ± 0.6 (n = 9) for 2-aminobenzimidazole and 8.3 ± 2.2 (n = 5) for Ru-1199. Since the effect on the sustained ASIC3-mediated currents was stronger than on the peak current, the plato/peak ratio of ASIC3 responses increased from 0.12 ± 0.05 (n = 15) in control to 0.23 ± 0.08 (n = 9) and 0.52 ± 0.03 (n = 5) in the presence of 2-aminobenzimidazole and Ru-1199, correspondingly.

**FIGURE 3:**
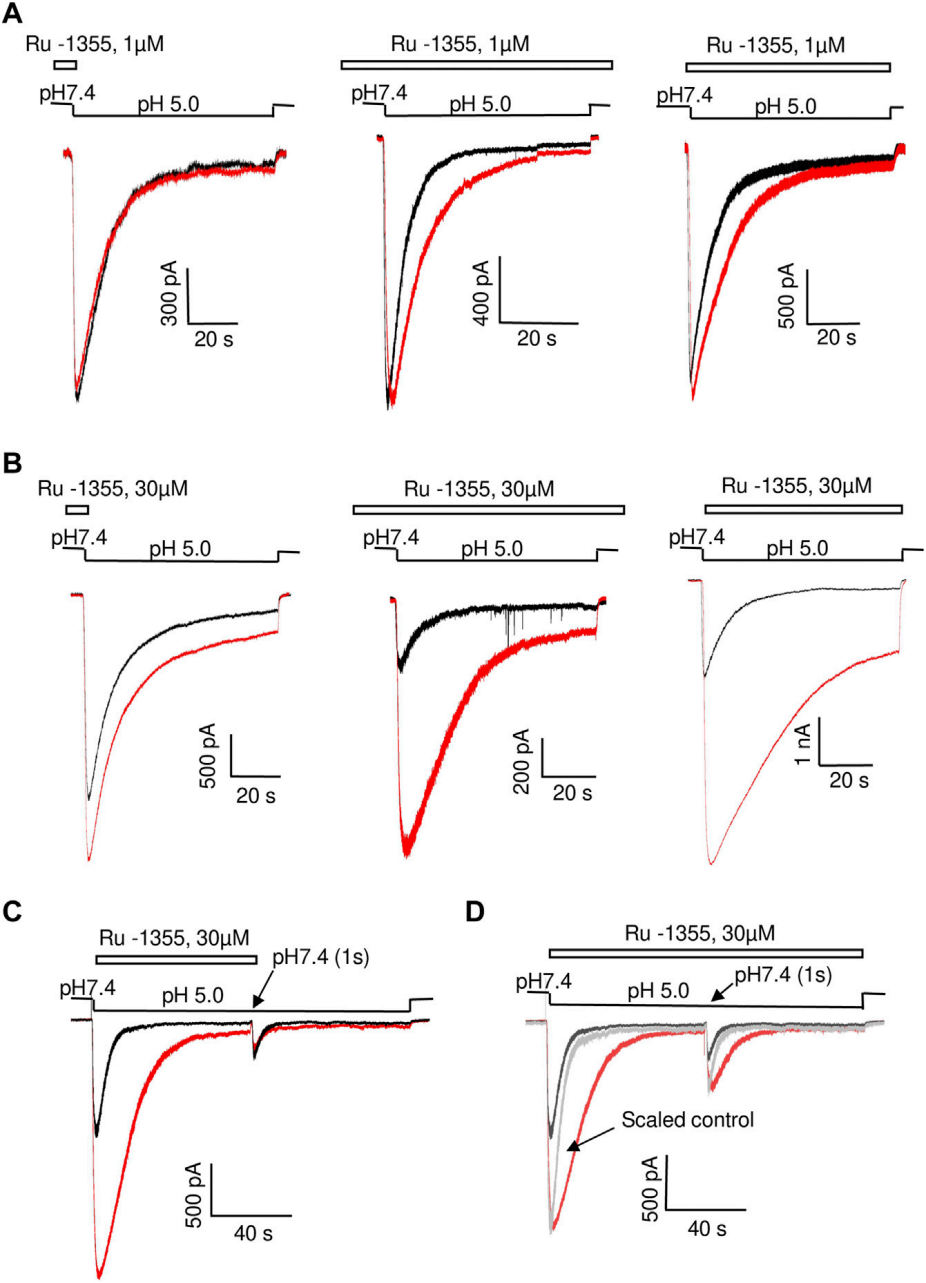
Comparison of Ru-1355 action on ASIC2a in different application protocols. Responses in the control condition are shown in black; responses in the presence of the drug are shown in red. Left panels show representative recordings in the experiments with Ru-1355 application only before activation. In the experiments shown in middle panels the compound was applied continuously. Right panels show the compound application simultaneously with the activating pH drops. **(A, B)**, action of 1 and 30 μM, correspondingly. The results are summarized in Table 2. **(C)**, fast recovery of Ru-1355 effect. In the paired-activation protocol (1 s interval) the amplitude of second ASIC2a response is smaller than the amplitude of the first response due to incomplete recovery from desensitization. If the first response is modified by 30 μM Ru-1355, the second response does not differ from the control one. **(D)**, Ru-1355 does on affect the recovery from desensitization. In the continuous presence of Ru-1355 (30 μM) amplitudes of the first and second response are equally increased. Scaled control response (gray) matches both amplitudes but the responses in the presence of Ru-1355 are much slower.

The experiments described above were performed with a co-application protocol, in which the drugs were applied simultaneously with acidifications. If the kinetics of the drug action is slow, the effect on the peak current may be significantly underestimated. Therefore, we tested the kinetics by comparing Ru-1355 action using three different application protocols (co-application, continuous application, and application only before activation). The results are presented in Table 2 and illustrated by Figures 3A, B. The effects of Ru-1355 (1 and 30 μM) on the peak and sustained components of the response and decay time constant were the same following the co-application or continuous application protocols (ANOVA with Tukey’s *post hoc* test, *p* > 0.05). In the case of application only before activation 1 μM Ru-1355 was virtually ineffective except weak potentiation of the sustained current. 30 μM caused the amplitude potentiation and decay slowing even in the case of application only before activation, but the effects were weaker than with other application protocols. Thus, the kinetics of the drug’s action were fast that confirmed the validity of using the co-application protocol in further experiments.

**TABLE 2:** Effects of Ru-1355 (drug vs. control) on ASIC2a response in different application protocols.

Application	Ru-1355 1 μM			Ru-1355 30 μM		
	Peak current	Sustained current	Decay constant	Peak current	Sustained current	Decay constant
Together with Activation (I)	1.00 ± 0.05 n = 6 (-)^a^	1.47 ± 0.14 n = 6 (+)	1.70 ± 0.33 n = 6 (+)	2.06 ± 0.53 n = 5 (-)	7.92 ± 1.02 n = 5 (+)	4.06 ± 0.71 n = 5 (+)
Continuously (II)	1.00 ± 0.03 n = 5 (-)	1.41 ± 0.15 n = 5 (+)	1.83 ± 0.09 n = 5 (+)	2.56 ± 0.76 n = 7 (+)	6.14 ± 2.27 n = 7 (+)	3.72 ± 1.43 n = 5 (+)
Before activation (III)	0.99 ± 0.03 n = 5	1.14 ± 0.07 n = 5	1.03 ± 0.03 n = 5	1.20 ± 0.11 n = 6	1.93 ± 0.25 n = 6	1.22 ± 0.14 n = 6

^a^- symbols in parentheses reflect the difference with the results of the drug application before activation (III). ANOVA, with Tukey’s *post hoc* test. (+) indicates that the difference is significant at the level of 0.05; (-) the difference is non-significant.

All effects of the drugs were fully reversible during 30 s intervals between activations. To test the kinetics of recovery, we used paired ASIC2a activations by pH 5.0 with 1 s interval. In control experiments the ratio between the amplitudes of first and second responses was 2.8 ± 0.4 (n = 6) due to incomplete recovery from desensitization. If Ru-1355 (30 μM) was applied simultaneously with the first activation, the second response was not significantly affected (n = 7, *p* > 0.05, paired *t*-test, drug vs. control) (Figure 3C) suggesting fast kinetics of recovery.

Paired-activation protocol was also used to reveal possible effect of Ru-1355 on the recovery of ASIC2a from desensitization. In these experiments 30 μM Ru-1355 was applied continuously. Figure 3D demonstrates that in the presence of Ru-1355 the ratio between amplitudes of the first and second responses (2.6 ± 0.4, n = 7) does not differ significantly (*p* > 0.05 paired *t*-test) from the control value despite the fact the response decay is strongly slowed down. Thus, slowing of the response decay by Ru-1355 is not related to corresponding changes in the stability of desensitized state.

### 3.3 Action of Ru-1355 on native ASICs

**FIGURE 4:**
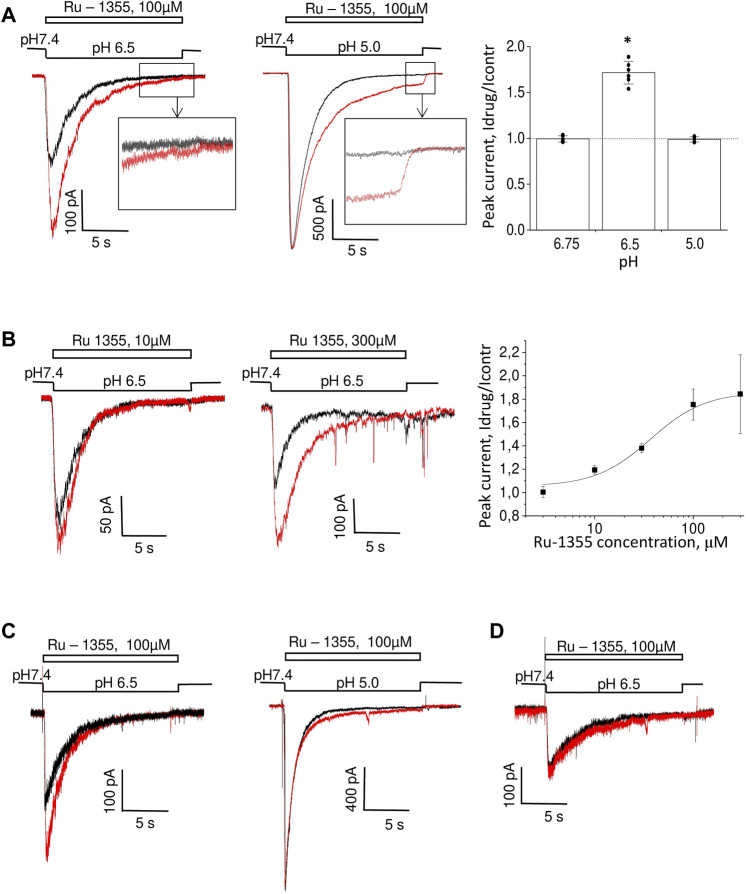
Action of Ru-1355 on native ASICs. Responses in the control condition are shown in black; responses in the presence of the drug are shown in red. **(A, B)**, action on cortex pyramidal neurons. **(A)**, pH dependence of the action. Ru-1355 causes a significant increase in the response to modest (pH 6.5) acidification. The amplitude of the response to strong (pH 5.0) acidification is not affected, but the drug causes a significant steady-state response. **(B)**, concentration dependence of the action. At 10 μM, the drug causes a subtle effect; at 300 μM it causes a saturating effect. **(C)** effect on striatal interneurons. The response is potentiated at pH 6.5 (left) but not at pH 5.0 (right). **(D)** responses in hippocampal pyramidal neurons are insensitive to Ru-1355.

The experiments were performed on pyramidal neurons from the second and third layers of the medial prefrontal cortex. Many previous studies have shown that the effect of some ASIC modulators depends on the activating pH value (Alijevic and Kellenberger, 2012; Tikhonova et al., 2015; Alijevic et al., 2018; Nikolaev et al., 2019). We measured the effect of Ru-1355 (100 μM) under conditions of weak (pH 6.75), medium (pH 6.5), and saturating (pH 5.0) activation (Figure 4A). The effect on the amplitude of the responses evoked by weak and strong acidifications was not significant (*p* > 0.005). At first glance, this type of pH dependence would appear to correspond to a parallel shift in the pH dependence of activation toward less acidic conditions. However, at pH 5.0, the application of Ru-1355 induced a significant steady-state response, which was absent in the control, despite the absence of an effect on the amplitude. This finding suggested a more complex mechanism of action.

We performed a concentration-dependence analysis for activation by pH 6.5. The results are shown in Figure 4B. Overall, 3 μM Ru-1355 was inactive (n = 5), 10 μM caused 1.19 ± 0.04 (n = 5) peak potentiation, and the highest tested concentration of 300 μM caused an effect of 1.84 ± 0.34 (n = 7). Fitting the concentration dependency using the Hill equation suggested the EC_50_ value of 47 ± 20 μM (n = 5–10) for activating pH 6.5.

For comparison, we tested action of 100 μM Ru-1355 on native ASICs in hippocampal CA1 pyramidal neurons and striatal interneurons (Figure 4C, D). At pH 6.5 the ASIC responses in striatal interneurons were significantly potentiated (Idrug/Icontr = 1.74 ± 0.17, n = 5) by 100 μM Ru-1355. As in the case of cortex pyramidal neurons, the peak potentiation became non-significant at pH 5.0 (Figure 4C). In contrast, the proton-activated current in hippocampal pyramidal neurons were not affected (Figure 4D).

### 3.4 Action of Ru-1355 on ASIC2a homomers and ASIC1a/2a heteromers

**FIGURE 5:**
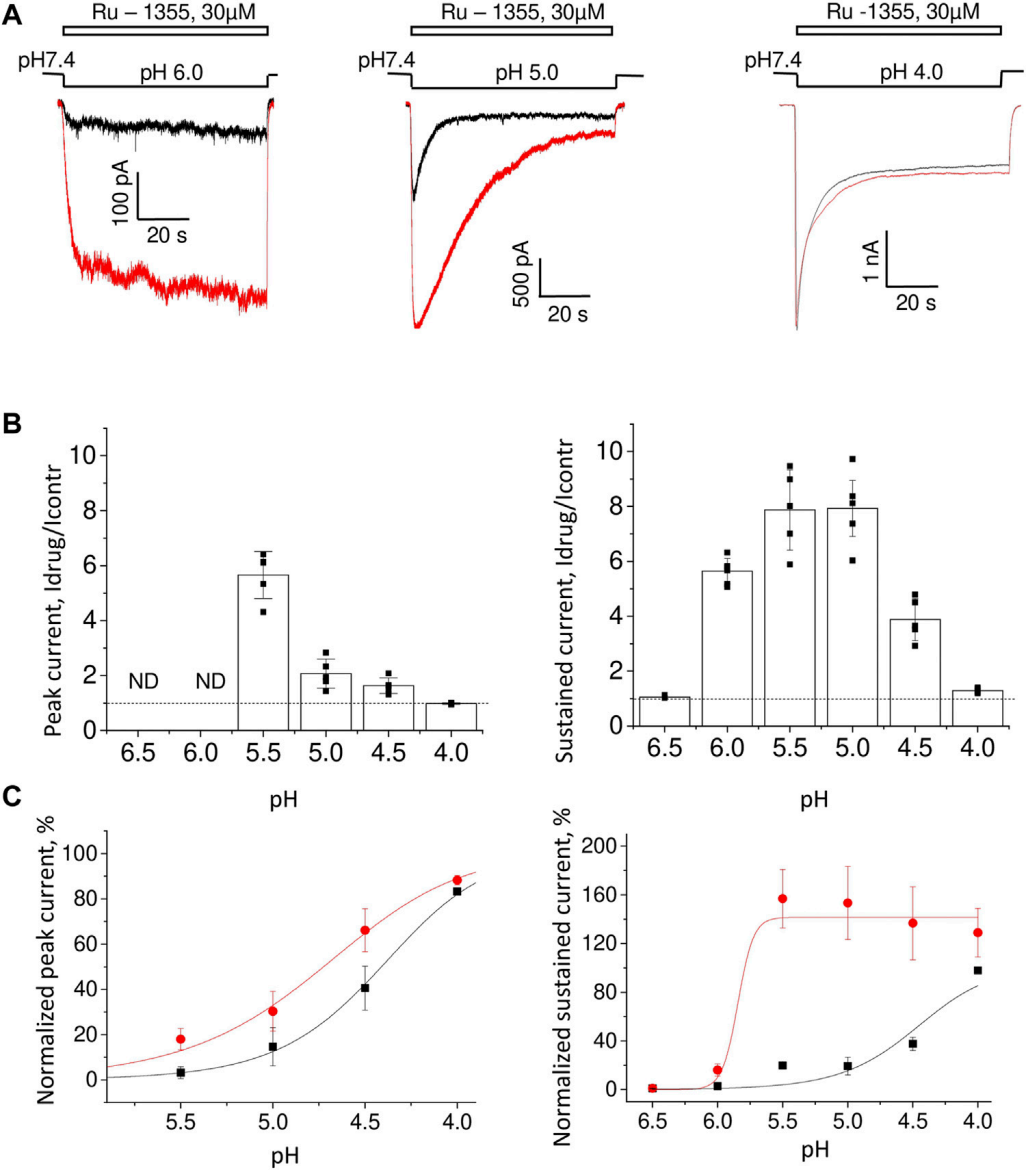
The pH dependence of Ru-1355 action on ASIC2a. **(A)**, representative responses evoked by different acidifications in the control condition (black) and in the presence of 30 μM Ru-1355 (red). Responses to pH 6.0 and 5.0 are strongly potentiated, whereas at pH 4.0, only minor potentiation of the sustained current is detected. **(B)**, summary data for the Ru-1355 effect on peak and sustained components of the response. The peak component is not pronounced at pH 6.5 and 6.0. In both cases, the drug becomes ineffective at strong acidifications. **(C)**, Ru-1355 produces an apparent shift of pH_50_ for both peak (left) and steady-state (right) components of the response.

As we had done for native ASICs, we first estimated the pH dependence of Ru-1355 action on the ASIC2a homomers. We used 30 μM Ru-1355 and applied it simultaneously with the activating pH. Representative recordings are shown in Figure 5A. The pH dependence of Ru-1355 (30 μM) action on the peak and sustained current is summarized in Figure 5B. At pH 6.5 and 6.0, the peak component was not seen for either the control response or the response in the presence of Ru-1355. The effect on the sustained current was 5.6 ± 0.5, n = 5 at pH = 6.0. At pH 5.5, the effects on the peak (Idrug/Icontr = 5.7 ± 0.9, n = 6) and sustained (Idrug/Icontr = 7.9 ± 1.5, n = 8) components were maximal. At activating pH 4.0 Ru-1355 had no effect on the peak component, and only a rather small effect on the sustained component (1.29 ± 0.09, n = 5). The decay time constant at pH 5.0 activation was increased from 5.2 ± 0.7 s (n = 7) in the control recordings to 21.1 ± 3.7 s (n = 5) in the presence of 30 μM Ru-1355. Unequal action of Ru-1355 on peak and steady-state components of the ASIC2a response resulted in decrease of the plato/peak ratio. At pH 5.0 this parameter increased from 0.08 ± 0.04 (n = 72) in control to 0.22 ± 0.11 (n = 8).

The pH-dependence of Ru-1355 action on the peak and sustained components of the response (Figure 5B) would apparently agree with a shift in the activation toward less acidic values (Figure 5C). For the peak current Ru-1355 increased the pH_50_ from 4.49 ± 0.16 (n = 5–7) in control to 4.86 ± 0.17 (n = 5–7). For the sustained current the pH_50_ was increased from 4.41 ± 0.12 (n = 5–7) in control to 5.8 ± 0.2 (n = 5–7). However, this simple suggestion does not explain the shape of the response at pH 6.0 (Figure 5A). The control response to a low agonist concentration did not demonstrate a pronounced peak component, because the channels open and desensitize asynchronically. In the case of potentiation due to the activation shift we can expect the response shape, which is typical for higher agonist concentrations (e.g., for pH 5.0). However, in our experiments, the response to pH 6.0 in the presence of Ru-1355 still had no peak component. Thus, Ru-1355 is unlikely to simply shift the pH dependence of ASIC2a activation.

**FIGURE 6:**
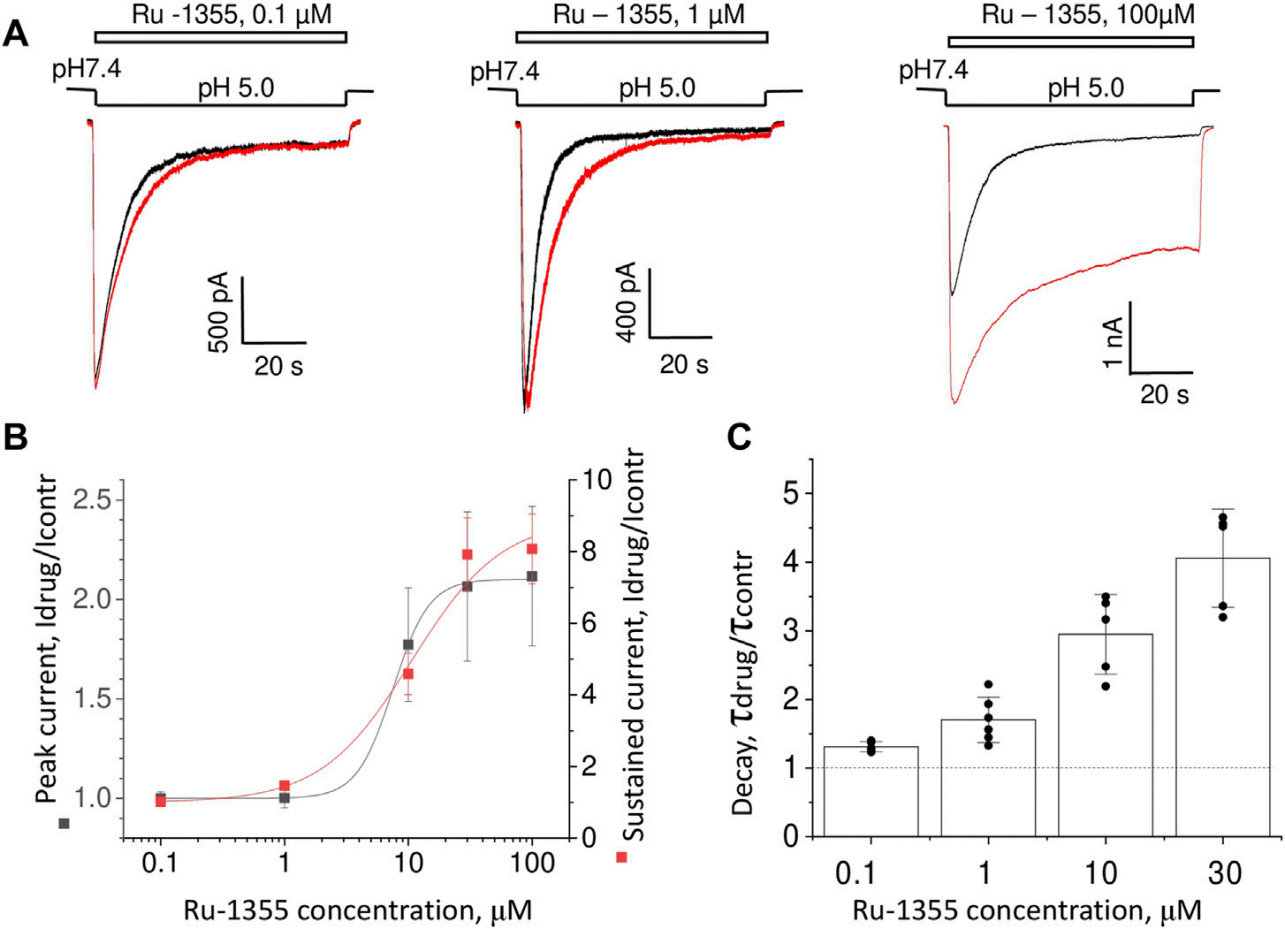
Concentration dependence of Ru-1355 action on ASIC2a. **(A)**, representative responses in the control condition (black) and in the presence of different concentrations of Ru-1355 (red). **(B, C)**, summary of the concentration dependence of the Ru-1355 effect on the peak and steady-state components **(B)** and on the decay time constant **(C)**. All three characteristics are affected with different concentration dependencies.

The concentration dependence of Ru-1355 action was estimated at pH 5.0. Figure 6A shows that Ru-1355 at 0.1 and 1 μM affected only the desensitization kinetics of ASIC2a. The effect on the peak component amplitude developed between 3 and 30 μM, whereas the effect on the sustained component was not saturated, even at 100 μM. Fitting of the concentration dependencies using the Hill equation suggested the EC_50_ value of 7.52 ± 0.33 μM (n = 5–7) for the peak component amplitude and 10.6 ± 2.9 μM (n = 5–7) for the sustained component (Figure 6B). Note that the sustained component was much more strongly potentiated than the peak component. As a result, the plato/peak ratio of the responses increased from 0.08 ± 0.04 (n = 72) in control to 0.36 ± 0.09 (n = 14) in the presence of 100 μM Ru-1355. A summary of the Ru-1355 action on the desensitization kinetics is given in Figure 6C. The strongest effect was observed at 30 μM (τdrug/τcontr = 4.1 ± 0.7, n = 5). The influence of higher concentrations on the decay kinetics was difficult to estimate (see section 3.5). A non-trivial conclusion from the analysis is that Ru-1355 affects three characteristics of the response (peak and sustained amplitudes and desensitization kinetics) with unequal dependences on the concentration.

**FIGURE 7:**
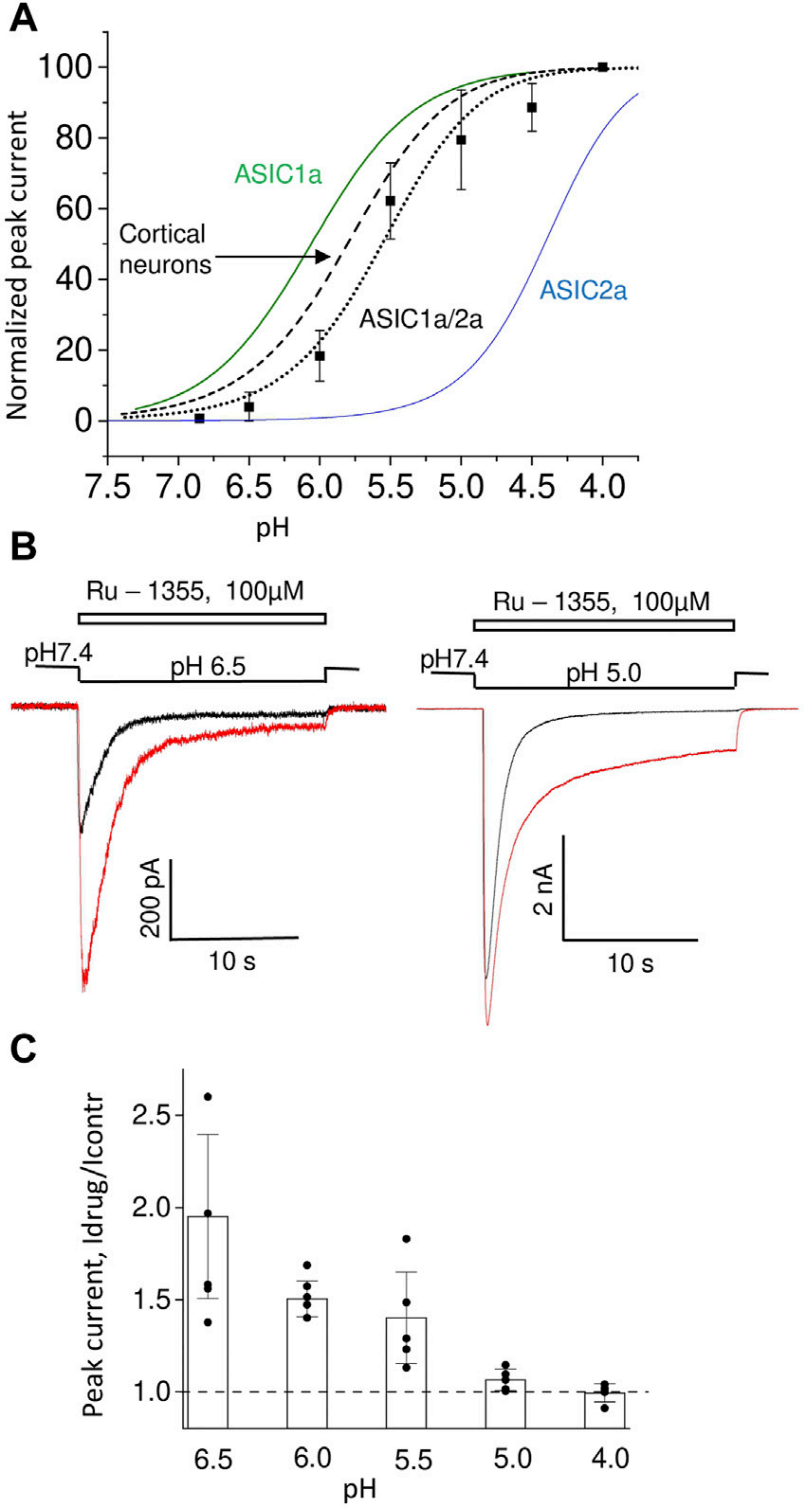
Action of Ru-1355 on ASIC1a/ASIC2a. **(A)**, pH dependence of peak current amplitudes. For comparison, the pH dependencies for homomeric ASIC1a and ASIC2a are presented. The data on native ASICs in cortical pyramidal neurons are taken from (Tikhonova and Barygin, 2019). ASIC1a data are from Nikolaev et al. (2019). ASIC2a data are from Figure 5C of the present work. **(B)**, representative responses at pH 6.5 (left) and pH 5.0 (right) in control (black) and in the presence of 100 μM Ru-1355 (red). The peak component is strongly potentiated at pH 6.5 but not at pH 5.0. The sustained component is potentiated in both cases. **(C)**, pH dependence of Ru-1355 action.

The effects of Ru-1355 were most pronounced for ASIC2a and least pronounced for ASIC1a, while the effect of native ASICs was intermediate. The obvious possibility is that native ASICs represents ASIC2a-containing heteromers. Therefore, we studied Ru-1355 action on CHO cells co-transfected with ASIC1a and ASIC2a plasmids (Figure 7). The pH dependence of activation of ASIC1a/ASIC2a heteromers is intermediate between corresponding homomeric channels with pH_50_ value of 5.6 ± 0.2 (Figure 7A). This value is close to the pH_50_ values (5.8–6.1) obtained for native ASICs in rat brain neurons (Tikhonova and Barygin, 2019). The responses demonstrated the pronounced peak component and very small steady-state component. In some cells the steady-state component was not detected. 100 μM Ru-1355 caused modest potentiation of peak component and strong potentiation of the steady-state component (Figure 7B). If sustained component of the response was not detected in control, it appeared in the presence of Ru-1355. The response decay became much slower in the presence of Ru-1355. Peak potentiation was pH dependent being the most pronounced at weak acidifications (Figure 7C). At strong acidification the action on the peak current became non-significant, whereas strong potentiation of the sustained current remained pronounced.

At pH 6.5 100 μM Ru-1355 caused 1.75 ± 0.13 (n = 10), 1.74 ± 0.17 (n = 5) and 1.9 ± 0.4 (n = 7) potentiation of native ASICs in cortex pyramidal neurons, striatal interneurons and ASIC1a/2a recombinant receptors, correspondingly. No significant differences were found between these values (unpaired t-tests, *p* > 0.005). Thus, the Ru-1355 action on ASIC1a/2a heteromers was very similar to the action on native ASICs in rat brain neurons. This action was significantly weaker than the ASIC2a potentiation, which under similar activation conditions (pH 5.5) was 5.7 ± 0.9 (n = 6) for 30 μM concentration.

### 3.5 Atypical responses of ASIC2a in the presence of Ru-1355 and Ru-1199

**FIGURE 8:**
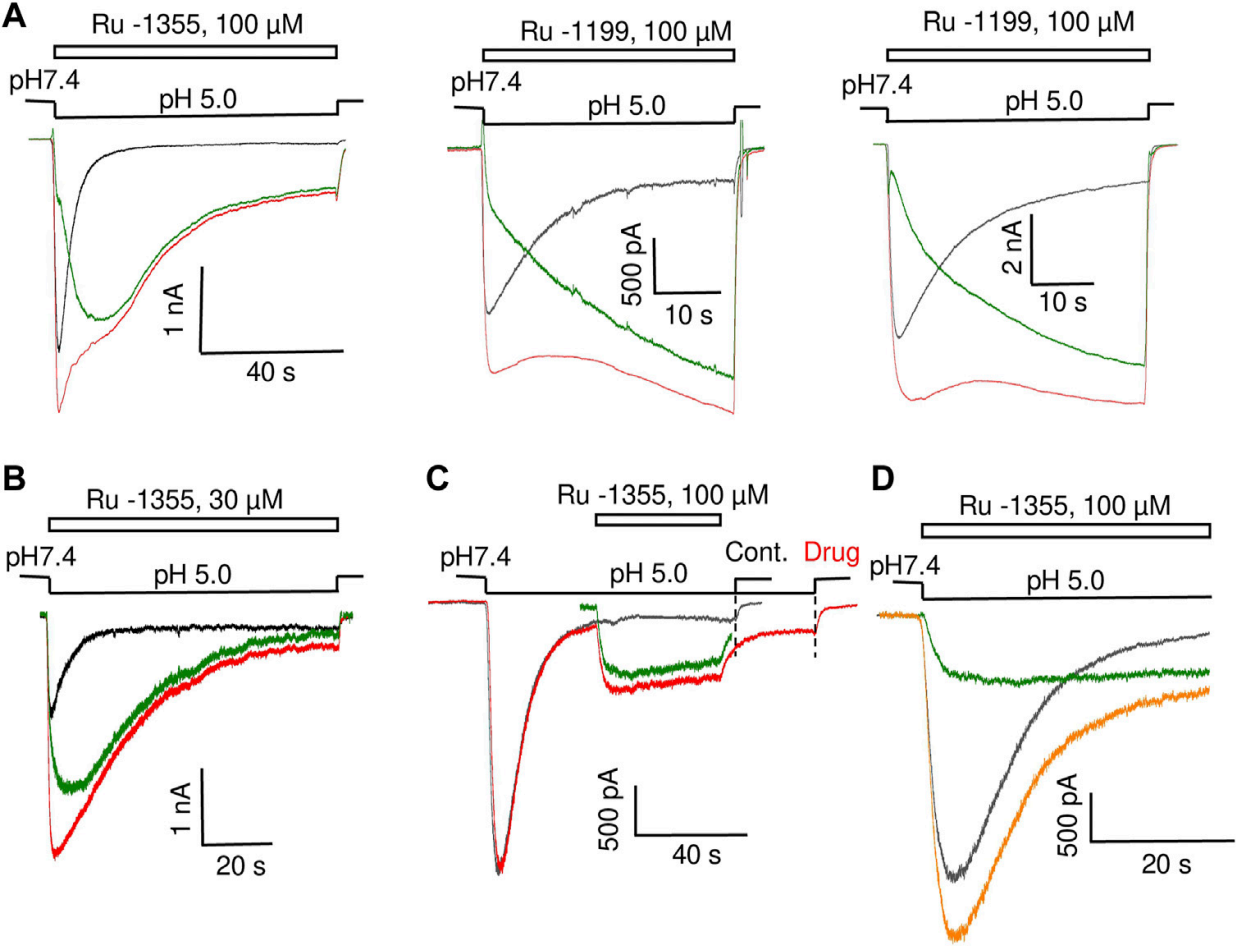
Additional ASIC2a currents evoked by Ru-1355 and Ru-1199. **(A)**, atypical ASIC2a responses in the presence of Ru-1355 or Ru-1199 (red) vs. the control response (black). Subtraction of the responses is shown in green. The result looks like an additional response, with slow kinetics of activation and desensitization. **(B)**, in the case of responses with mono-exponential decay, the result of subtraction is also seen as extra responses with slow kinetics. **(C)**, application of 100 μM Ru-1355 after response equilibration causes an extra response with slow kinetics (green). **(D)**, the extra response shown in C is computationally added at the beginning of the control response. The result of summation (orange) resembles the response in the presence of Ru-1355.

Application of Ru-1355 at concentrations of 100 μM and application of Ru-1199 at a concentration of 10 μM caused an ASIC2a response with an atypical shape (Figure 8A). Instead of a monoexponential decay, the responses resembled the control responses that are followed by additional currents with slow development and slow desensitization. 30 μM Ru-1199 induced a biphasic response, in which the fast and slow components were separated by a minimum. Subtracting the control response from the modified response visualized this component (Figure 8A). This behavior cannot be explained by a simple mechanism in which Ru-1355 affects the activation and desensitization of ASIC2a. A similar subtraction demonstrates that, even for responses with a classical shape with exponential decay (Figure 8B), the difference between the control response and the response in the presence of Ru-1355 can also be represented as an additional slow component. Atypical effects of Ru-1355 and Ru-1199 were completely reversible (data not shown). To prove that these unusual responses were due to ASIC2a modulation, we performed additional control experiments that demonstrated the absence of any responses to 100 μM Ru-1199 at pH 7.4 in the same cells.

The presence of a sustained component in the control ASIC2a response allows the application of drugs after the response equilibration. Figure 8C shows the results. The application of 100 μM Ru-1355 caused a reversible additional current (Idrug/Icontr = 2.3 ± 0.5, n = 6) with slow desensitization. We extracted this current *in silico* and added it to the beginning of the control response (see Figure 8D). The sum noticeably resembles the currents in the presence of Ru-1355, which were characterized above. Thus, our data allows to propose that Ru-1355 and Ru-1199 induce an additional component of the ASIC2a response with slow kinetics. Certainly, this hypothesis needs validation.

### 3.6 Diminazene discriminates open states with fast and slow kinetics in ASIC2a and ASIC3

Fast and slow response components may correspond to different open states. If this is the case, these different open states may show different sensitivities to drugs that block the pore. We tested this idea by studying the action of diminazene (Chen et al., 2010a; Chen et al., 2010b).

**FIGURE 9:**
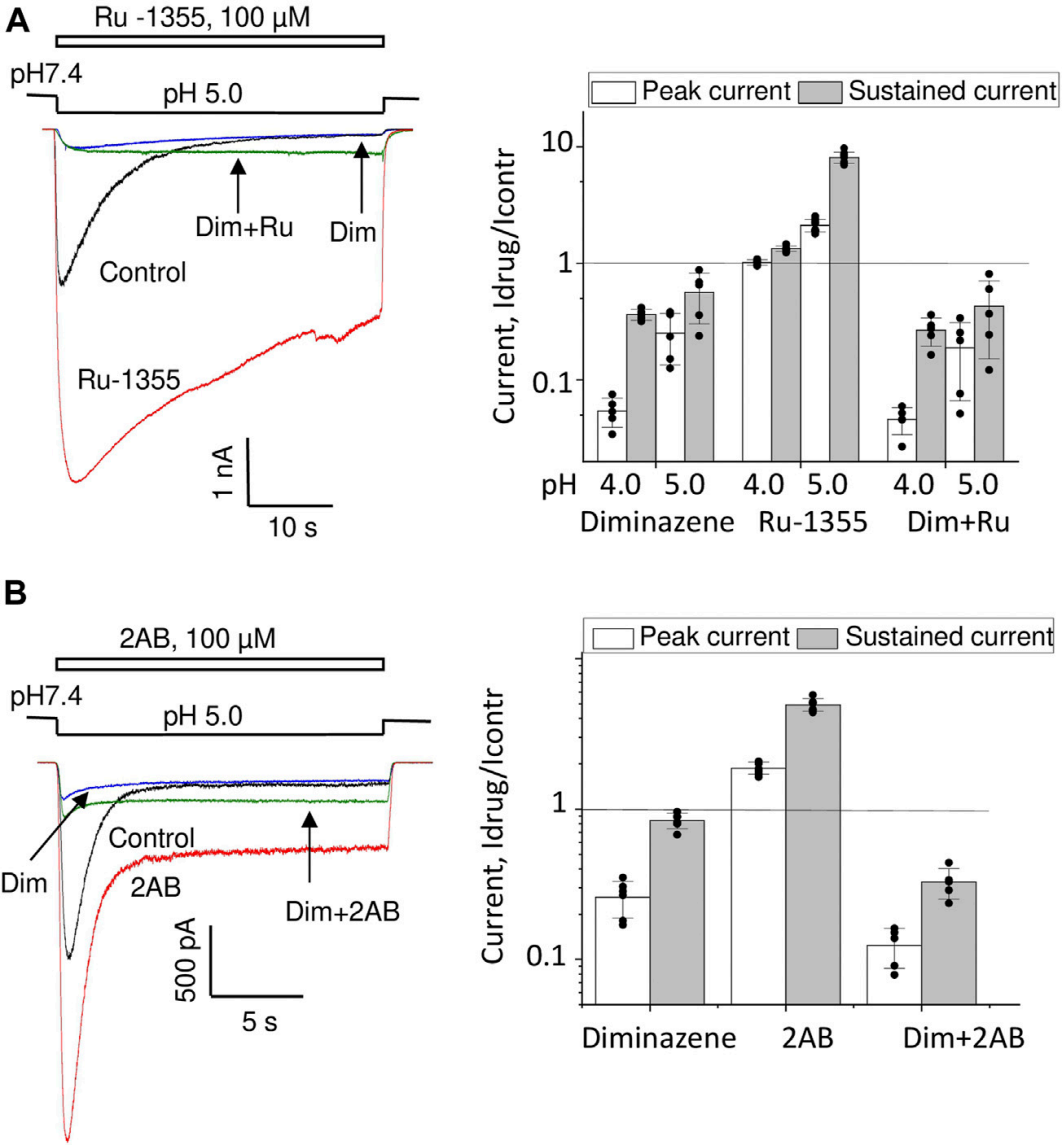
Action of diminazene (70 μM) on ASIC2a **(A)** and ASIC3 **(B)** in the control condition and in the presence of potentiators. Responses in the control condition are shown in black, in the presence of potentiators are shown in red, in the presence of diminazene are shown in blue, and in the presence of both potentiators and diminazene are shown in green. In all cases, the peak component of the response is strongly inhibited, whereas the effect on the sustained component is much weaker. The right panels summarize the effects on the peak and sustained components.

Effect of diminazene on ASIC2a-mediated currents was studied at two values of activating pH; at 5.0 only about 20% of maximal response is seen, whereas pH 4.0 causes saturating activation (see Figure 5C). At pH 5.0 70 μM diminazene caused 75 ± 12% (n = 6) inhibition of the peak ASIC2a response and only a 44 ± 24% (n = 6) inhibition of the sustained response (Figure 9A). At pH 4.0 the difference was even more pronounced, diminazene caused 94 ± 2% (n = 7) and 64 ± 4% (n = 6) inhibition of the peak and sustained response components, correspondingly. The same was true for the responses modified by 100 μM Ru-1355; at pH 5.0 peak and sustained currents were inhibited by 81 ± 13% (n = 5) and 57 ± 28% (n = 5), correspondingly. At pH 4.0 the corresponding values were 95 ± 2% (n = 5) and 73 ± 9% (n = 5).

Thus, under all conditions for both ASIC2a response either in control and in the presence of potentiating drugs the sustained currents were inhibited weaker than the peak ones. This finding prompted us to study the effects of diminazene on the peak and sustained components of ASIC3 responses in control and in the presence of 2-aminobenzimidazole. In control the peak current was inhibited by 74 ± 8% (n = 6) by 70 μM diminazene, whereas the sustained current was blocked by only 16 ± 10% (n = 6) (Figure 9B). The same was true for the ASIC3 response potentiated by 100 μM 2-aminobenzimidazole; at 70 μM concentration, diminazene practically eliminated (88 ± 4%, n = 5) the peak component of the ASIC3 response but the sustained component was only blocked by 67 ± 7% (n = 5).

Different action of diminazene on the peak and sustained components of ASIC2a and ASIC3 responses either in control and in the presence of potentiating drugs suggest that these components are mediated by structurally distinct open states.

## 4 Discussion

In this study, we present new ASIC-targeting compounds. Among the compounds studied only Ru-1270 in 100 μM concentration caused modest inhibition of native ASICs. At low micromolar concentrations Ru-1355 selectively potentiates homomeric ASIC2a. The drug also potentiates native ASICs, albeit with lower activity. Analysis of Ru-1355 action on ASIC1a/ASIC2a heteromers suggests that moderate potentiation of native ASICs likely reflects their heteromeric subunit composition. Sensitivities of ASIC1b and ASIC2b-containing heteromers to the 2-aminobenzimidazole derivatives were not studied, the latter are present in the central nervous system and can also contribute to the intermediate action of the drugs on native channels. Notably, selective potentiators of ASIC1a homomers are usually not active on native ASICs. One such example is histamine (Nagaeva et al., 2016b; Barygin et al., 2017). Conversely, selective ASIC2a potentiators, such as IEM-1921 and IEM-2117, are also active on native ASICs (Tikhonova et al., 2015). Ru-1355 also follows this pattern. Ru-1199 is more potent ASIC2a potentiator, at 3 μM it does not affect other ASICs. High activity and selectivity of these drug is useful for physiological studies of ASICs.

The action of Ru-1355 was previously studied *in vitro* and in animal models (Spasov et al., 2016). Ru-1355 had very high inhibitory activity against NHE-1; therefore, it is probably a multitarget substance with systemic effects. Interestingly, Ru-1355 causes NHE-1 inhibition and ASIC2a potentiation, while amiloride blocks both NHE-1 (Benos, 1982) and ASICs.

Analysis of the action of Ru-1355 and Ru-1199 demonstrated that simple effects on activation and desensitization cannot fit the experimental data. At low concentrations the compounds caused amplitude increase and decay slowing down. Concentration and pH dependencies of Ru-1355 action on peak responses, steady-state responses and responses decay were different. At high concentrations Ru-1199 and Ru-1355 caused atypical ASIC2a responses. Instead of an exponential decay, the responses in the presence of the drugs resembled the control transient responses that are followed by additional currents with slow development and slow desensitization. It looks like the compounds activate or potentiate a specific ASIC2a open state with slow activation and desensitization kinetics. The effects of low concentrations on the response amplitude and decay can also be explained in this way. To test this hypothesis, we used the ASIC pore blocker diminazene. The slow currents induced by the drugs were found to be weakly sensitive to diminazene in contrast to the peak components of the responses, which were strongly inhibited. This finding evidences against the simple explanations of the drugs action *via* modulation of channel activation and (or) desensitization. Surprisingly, even in control experiments diminazene blocked peak components of ASIC2a and ASIC3 responses much stronger than the sustained ones. This result agrees with the diminazene effect on ASIC3 observed by (Lee et al., 2018).

In some previous studies it has been observed that the ASIC3 response does not monotonously decay from the peak to the steady-state level but instead demonstrates a pronounced minimum between the components (Hesselager et al., 2004; Osmakov et al., 2018). Heteromers ASIC2a/ASIC3 also demonstrate this type of response (Hesselager et al., 2004). Some drugs can induce only the slow component of the ASIC3 response (Osmakov et al., 2018). Although ASIC1a does not mediate sustained currents, several Cys mutants in the palm domain demonstrate non-desensitizing components of the response (Roy et al., 2013). This component appears and (or) increases after applying of MTSET. Combined mutations (Vullo et al., 2017) produced various types of ASIC1a responses with different combinations of peak and sustained components. It has been demonstrated that sustained currents of mutant ASIC1a differ from the peak currents not only by kinetics and desensitization but also in i) ionic selectivity, ii) sensitivity to the pore blocker amiloride and iii) pH dependence of activation (Vullo et al., 2017).

Thus, more data increasingly suggest that stable ASIC-mediated currents do not reflect incomplete desensitization but instead represent a specific open state. In view of this hypothesis the ASIC1a lack this slow-kinetics open state and shows only the transient response. A slow-kinetics open state is detectable for ASIC2a and is especially pronounced in ASIC3. According to our data, the primary mechanism of action of 2-aminobenzimidazole derivatives is to favor this slow-kinetics open state in ASIC2a. Thus, activation of the slow open state can be controlled not only by ASIC sequences, but also by pharmacological agents. Note that MitTX activates slow currents trough ASIC1a and ASIC1b and strong potentiation of ASIC2a responses is accompanied by slowing of response decay (Bohlen et al., 2011).

The hypothesis about existence of two distinct open states need further validation. Besides pharmacological discrimination that employs selective drugs, single-channel analysis can provide valuable information on the opening probability, open time, conductance, and response to the drugs in each of the multiple states. Available data (Zhang and Canessa, 2001; 2002) demonstrate complex multi-modal ASIC activity. At present, it would be premature to speculate about the exact relationships between fast and slow ASIC open states and to propose comprehensive kinetic models.

## Data Availability

The raw data supporting the conclusions of this article will be made available by the authors, without undue reservation.
